# Peripheral blood cytokines as potential diagnostic biomarkers of suicidal ideation in patients with first-episode drug-naïve major depressive disorder

**DOI:** 10.3389/fpubh.2022.1021309

**Published:** 2022-11-07

**Authors:** Yayun Xu, Jun Liang, Wenfan Gao, Yanhong Sun, Yuanyuan Zhang, Feng Shan, Jinfang Ge, Qingrong Xia

**Affiliations:** ^1^Department of Epidemiology and Biostatistics, School of Public Health, Anhui Medical University, Hefei, China; ^2^Inflammation and Immune Mediated Diseases Laboratory of Anhui Province, Anhui Institute of Innovative Drugs, Anhui Medical University, Hefei, China; ^3^The Key Laboratory of Anti-Inflammatory and Immune Medicines, Ministry of Education, Hefei, China; ^4^Affiliated Psychological Hospital of Anhui Medical University, Hefei, China; ^5^Department of Pharmacy, Hefei Fourth People's Hospital, Hefei, China; ^6^Psychopharmacology Research Laboratory, Anhui Mental Health Center, Hefei, China; ^7^Anhui Clinical Research Center for Mental Disorders, Hefei, China; ^8^School of Pharmacy, Anhui Medical University, Hefei, China

**Keywords:** cytokines, biomarker, diagnosis, serum, major depressive disorder, suicidal ideation

## Abstract

**Objective:**

Major Depressive Disorder (MDD) is a leading cause of disability, with a high risk of suicidal ideation (SI). Few studies have evaluated the potential of multiple cytokines as biomarkers for SI in patients with MDD. In the present study, we examined the serum levels of multiple cytokines in patients with first-episode drug-naïve MDD, with the aim to discover and identify serum cytokines-based biomarkers for identification of SI in MDD.

**Methods:**

A total of 55 patients with first-episode drug-naïve MDD were enrolled and divided into two groups: 26 MDD patients without SI and 29 MDD patients with SI. Beck Scale for Suicide Ideation was used to estimate SI. A total of 37 cytokines were measured using Multiplex Luminex Assays. The levels of serum cytokines between MDD patients without SI and MDD patients with SI were compared and diagnostic values of different cytokines were evaluated using the receiver operating characteristic (ROC) curve method for discriminating MDD patients with SI from MDD patients without SI. The relationship between the group and the abnormal cytokines were investigated in multiple linear regression models, with adjustments for age, gender, BMI, smoking, and Hamilton Depression Rating Scale-24 (HAMD-24) scores.

**Results:**

The levels of CCL26 and VEGF in MDD patients with SI were significantly lower than those in MDD patients without SI (all *P* < 0.05). On the contrary, the levels of IL-17C, CXCL10, and TNF-β in MDD patients with SI were significantly higher than those in MDD patients without SI (all *P* < 0.05). Moreover, the results of multiple linear regression revealed that group was a significant independent predictor of serum IL-17C, CCL-26, VEGF, and TNF-β levels (all *P* < 0.05). In terms of CXC10, group was also likely to be a significant independent predictor (β = 0.257, *P* = 0.063). Furthermore, the AUC values of IL-17C and TNF-β were 0.728 and 0.732, respectively. Additionally, a combined panel of IL-17C and TNF-β achieved a high accuracy in discriminating MDD patients with SI from MDD patients without SI (AUC = 0.848, sensitivity = 75.9%, specificity = 72.7%).

**Conclusions:**

These results suggested that circulating IL-17C and TNF-β may hold promise in the discovery of biomarkers for identification of SI in MDD.

## Introduction

Major Depressive Disorder (MDD), one of the most common psychiatric disorders, is associated with increased risk of suicidal ideation (SI), suicide attempts and completed suicide (1, 2). According to the World Health Organization (WHO), MDD affects 300 million people globally. It has been reported that 58% of MDD patients have SI and 15% have attempted suicide (3, 4). Due to its high morbidity, mortality and disability rate, MDD places a very heavy burden on families and society. Currently, the diagnosis of depression and SI primarily depends on subjective symptoms/manifestations, with uncertainties as high as 40% as a consequence (5). Therefore, the use of objective biomarkers to identify subgroups at high risk of suicide in patients with MDD has important clinical implications for suicide prevention in the clinical setting.

Several lines of evidence indicate a close relationship between cytokines and MDD. It has been demonstrated that patients with MDD manifested elevated peripheral levels of interleukin-6 (IL-6) and C-reactive protein (CRP) in a cumulative meta-analysis (6). Moreover, increased levels of peripheral CRP and IL-6 were associated with an increased risk of depressive episodes and subsequent development of depressive symptoms (7, 8). Furthermore, depressed patients who are resistant to conventional antidepressants showed higher peripheral blood levels of pro-inflammatory cytokines (9, 10). Additionally, alterations in peripheral cytokine levels were associated with antidepressant treatment outcomes in MDD (11). These findings suggest that peripheral cytokines are implicated in the pathophysiology of depression and may hold significant promise as potential biomarkers for identification of SI in MDD.

Although no objective biomarkers of SI risk currently exist, numerous evidence has suggested several biomarkers that hold promise as predictive indicators of SI. A recent review summarized the last 5 years of research into suicide-associated biomarkers and found that the serotonergic system, inflammation, hypothalamic-pituitary-adrenal axis, lipids, and endocannabinoids emerged as the most promising diagnostic, predictive, and therapeutic indicators (12). Moreover, another meta-analysis evaluated the relationship between blood hormone levels and suicidal behavior (13). The results suggested that blood thyrotropin stimulating hormone (TSH), leptin and dehydroepiandrosterone sulfate (DHEAS) levels were associated with suicide attempts, and progesterone levels were associated with SI (13). Furthermore, biochemical indicators including alpha 1-antitrypsin (AAT), transferring, high-density lipoprotein cholesterol, and apolipoprotein A1 were identified to be potential biomarkers of SI (14). It is noteworthy that the biological mechanisms of suicidality may be related to stress, inflammation and apoptosis (15). Additionally, several proinflammatory cytokines have been reported to be related to suicidality. Specifically, it has been shown that the serum IL-1β level was lowest in suicide attempters, differentiating them from suicide ideators or healthy controls (16). A systematic review found that blood IL-2, IL-6, IL-8, tumor necrosis factor-α (TNF-α), and vascular endothelial growth factor (VEGF) levels were altered in patients with suicidal behavior (17). In MDD patients, increased baseline TNF-α might predict changes of SI intensity (18). Taken together, few studies have evaluated the potential of multiple cytokines as biomarkers for SI in patients with MDD.

In the present study, we examined the serum levels of multiple cytokines in patients with MDD, with the aim to discover and identify serum cytokines-based biomarkers in discriminating MDD patients with SI from MDD patients without SI. A total of 37 cytokines in patients with MDD were measured and Beck Scale for Suicide Ideation was used to estimate suicidal ideation. Subsequently, the levels of serum cytokines between MDD patients without SI and MDD patients with SI were compared and diagnostic values of different cytokines were evaluated using the receiver operating characteristic (ROC) curve method for discriminating MDD patients with SI from MDD patients without SI.

## Materials and methods

### Study design and participants

This study was conducted at Anhui Mental Health Center between August 2020 and June 2022. Fifty-six patients with first-episode drug-naïve MDD were diagnosed by trained psychiatrists according to the Diagnostic and Statistical Manual for Psychiatric Disorders-Fifth Version (DSM-V). Common criteria for patient inclusion were as follows: (1) being between the ages of 18–65; (2) meeting DSM-V criteria for depression; (3) Hamilton Depression Rating Scale-24 (HAMD-24) scores higher than 20; and (4) receiving no treatment with antidepressants, anti-inflammatory agents or other psychotropic drugs in the previous 3 months. Common criteria for patient exclusion were as follows: (1) current or lifetime history of major neurological disorder or other psychiatric disorders; (2) current or lifetime history of substance abuse other than tobacco; (3) chronic infections, inflammatory, or immune disorders; (4) currently receiving anti-inflammatory treatment. A full medical examination and a detailed medical history inquiry were recorded to fulfill the inclusion/exclusion criteria. This procedure was approved by the ethics committee of the Anhui Mental Health Center (registration number HFSY-IRB-PJ-XQR-2020001) and was conducted according to the principles of the Declaration of Helsinki. Informed consent was obtained from all the participants.

### Clinical assessment

Suicidal ideation was estimated by the 19-item Beck Scale for Suicide Ideation (19). Individuals who scored > 0 on either item 4 or 5 were considered to be currently suicidal. Individuals who received a score of 0 for both these items were considered to be currently non-suicidal (20). In the present study, 55 MDD patients were divided into two groups accordingly: 26 MDD patients without SI and 29 MDD patients with SI. HAMD-24 was used to evaluate the severity of depressive symptoms in all participants.

### Blood sample collection and measurement of serum cytokines

The blood samples from the subjects were collected between 7:00 and 8:00 o'clock, centrifuged at 1,200 g for 10 min at 4°C. The supernatant was used as serum samples, which were maintained at−80°C until detection. The blood samples were collected at baseline before treatment. A total of 37 serum cytokines, including IL-1α (also called IL-1F1), IL-1β (also called IL-1F2), IL-1RA (also called IL-1F3), IL-2, IL-3, IL-4, IL-5, IL-6, IL-7, IL-8 (also called C-X-C motif chemokine ligand 8, CXCL8), IL-10, IL-12 (also called IL-23 p40), IL-12 p70, IL-13, IL-15, IL-16, IL-17C, IL-27, IL-31, C-C motif chemokine ligand 3 (CCL3; also called macrophage inflammatory protein 1α, MIP-1α), CCL4 (also called MIP-1β), CCL11 (also called eotaxin), CCL17 (also called thymus and activation regulated chemokine, TRAC), CCL26 (also called eotaxin-3), CXCL10 (also called interferon-inducible Protein 10, IP-10; cytokine responsive gene-2, CRG-2), vascular endothelial growth factor (VEGF), VEGF-C, VEGFR1 (also called Flt1), TNF-α, TNF-β (also called lymphotoxin), Tie-2, interferon-γ (IFN-γ), granulocyte-macrophage colony-stimulating factor (GM-CSF), fibroblast growth factor-basic (FGF basic, also called FGF2/bFGF), thymic stromal lymphopoietin (TSLP), intercellular cell adhesion molecule-1 (ICAM-1), and placenta growth factor (PIGF) were measured by the multiplex bead immunoassay (LXSAHM-10 and LXSAHM-27, R&D system for antibody detection, Shanghai Universal Biotech Co., Ltd) according to the manufacturer's instructions.

### Statistical analysis

Statistical analysis was calculated using SPSS (version 17.0; IBM Corp., Armonk, NY, USA). The data are shown as mean ± standard error of the mean (SEM), and the statistical significance was set at *P* < 0.05. The normality of continuous variable was evaluated using the Kolmogorov-Smirnov normality test. Normally distributed data were compared using the Student's *t*-test, whereas data following a non-normal distribution were analyzed using a non-parametric test (Mann-Whitney U test). Chi-squared test was used to determine the difference between the two groups with respect to sex, marital status, and smoking status. Two-group comparisons of the serum cytokines were performed using Student's *t*-test or Mann-Whitney U test and false discovery rate (FDR) adjustment using the Benjamini-Hochberg (BH) adjusted *P*-value, correcting for multiple testing. The relationship between the group and the abnormal cytokines were investigated in multiple linear regression models, with adjustments for age, gender, BMI, smoking, and HAMD-24 scores. The correlation between the severity of suicidal ideation (as measured by the 19-item Beck Scale for Suicide Ideation) and the cytokines identified as being elevated in MDD patients with SI was evaluated by Spearman's correlation test. The diagnostic performance of serum cytokines was estimated using the receiver operating characteristic (ROC) curve analysis and calculating the area under the curve (AUC) values in discriminating MDD patients with SI from MDD patients without SI.

## Results

### Demographic and clinical characteristics of the participants

Table 1 summarizes the demographic and clinical characteristics of MDD patients without SI and MDD patients with SI. There were no significant differences in age, sex BMI, years of education, marital status, smoking status, or HAMD scores between the two groups (Table 1).

**Table 1:** Demographic and clinical characteristics of MDD patients without SI and MDD patients with SI.

**Variables**	**MDD without SI**	**MDD with SI**	***t*/Z/*χ^2^***	***P***
Age	37.04 ± 2.85	35.52 ± 2.71	−0.574	0.566
Sex (Female/Male)	19/7	16/13	1.899	0.168
BMI (kg/m^2^)	22.24 ± 0.74	22.65 ± 0.58	−0.435	0.665
Education (years)	9.23 ± 1.09	9.83 ± 0.88	−0.431	0.668
Marital status (Yes/No)	16/10	15/14	0.537	0.464
Smoking status (Yes/No)	2/24	5/24	1.125	0.289
HAMD-24	33.58 ± 2.14	36.41 ± 1.50	−1.106	0.274

### Differences of cytokine levels in serum between MDD patients without SI and MDD patients with SI

As shown in Table 2, the levels of CCL26 and VEGF in MDD patients with SI were significantly lower than those in MDD patients without SI (all *P* < 0.05). On the contrary, the levels of IL-17C, CXCL10, and TNF-β in MDD patients with SI were significantly higher than those in MDD patients without SI (all *P* < 0.05). After applying the Benjamini-Hochberger correction for multiple measurements, none of these differences persisted (all *P* > 0.05).

**Table 2:** Comparison of serum cytokines between MDD patients without SI and MDD patients with SI.

**Variables (pg/ml)**	**MDD without SI**	**MDD with SI**	***t*/*Z***	***P***	**BH adj. *P*-value**
IL-1α (IL-1F1)	4.15 ± 0.86	4.45 ± 0.67	−0.941	0.347	0.713
IL-1β (IL-1F2)	10.97 ± 2.04	8.62 ± 1.23	−0.978	0.328	0.759
IL-1RA (IL-1F3)	1498.53 ± 314.79	856.69 ± 66.93	−1.863	0.062	0.328
IL-2	40.96 ± 7.44	29.92 ± 4.99	−1.139	0.255	0.726
IL-3	91.90 ± 63.54	27.45 ± 1.10	−0.609	0.542	0.802
IL-4	34.84 ± 6.85	33.95 ± 5.18	−0.034	0.973	1.000
IL-5	2.20 ± 0.30	2.04 ± 0.14	−0.431	0.667	0.851
IL-6	17.85 ± 4.67	5.92 ± 1.04	−1.431	0.153	0.566
IL-7	10.35 ± 1.22	10.38 ± 1.12	−0.008	0.993	0.993
IL-8 (CXCL8)	752.39 ± 171.23	376.43 ± 63.85	−1.619	0.105	0.486
IL-10	3.45 ± 0.88	3.58 ± 0.65	−0.285	0.775	0.896
IL-12 (IL-23 p40)	227.76 ± 18.08	232.22 ± 10.69	−0.793	0.428	0.720
IL-12 p70	44.86 ± 12.80	35.37 ± 6.243	−0.980	0.327	0.807
IL-13	264.30 ± 27.34	267.81 ± 17.26	−0.862	0.389	0.720
IL-15	4.12 ± 0.64	4.21 ± 0.46	−0.746	0.456	0.703
IL-16	193.44 ± 15.58	195.77 ± 39.44	−1.443	0.149	0.613
IL-17C	9.08 ± 0.80	11.95 ± 0.73	−2.840	0.005	0.093
IL-27	322.72 ± 31.56	312.40 ± 26.58	−0.178	0.859	0.935
IL-31	30.21 ± 1.86	27.49 ± 1.39	−0.928	0.353	0.687
CCL3 (MIP-1α)	924.76 ± 251.71	574.53 ± 110.94	−0.970	0.332	0.723
CCL4 (MIP-1β)	622.73 ± 143.81	476.27 ± 71.48	−0.270	0.787	0.882
CCL11 (Eotaxin)	107.08 ± 10.11	122.59 ± 9.91	−0.861	0.389	0.685
CCL17 (TRAC)	277.76 ± 18.24	377.85 ± 35.11	−1.946	0.052	0.321
CCL26 (Eotaxin-3)	15.95 ± 1.30	11.95 ± 0.92	−2.482	0.013	0.160
CXCL10 (IP-10/CRG-2)	17.18 ± 0.97	21.59 ± 1.65	−2.207	0.032	0.237
VEGF	133.14 ± 16.04	90.11 ± 8.72	−2.251	0.024	0.222
VEGF-C	1719.07 ± 117.59	1796.53 ± 115.33	−0.469	0.641	0.878
VEGFR1 (Flt1)	148.15 ± 9.95	165.36 ± 12.63	−1.054	0.297	0.785
TNF-α	7.04 ± 1.57	5.30 ± 1.00	−0.787	0.431	0.693
TNF-β (Lymphotoxin)	2.29 ± 0.16	3.76 ± 0.64	−2.930	0.003	0.111
Tie-2	16686.08 ± 1547.17	16566.31 ± 1869.55	−0.287	0.774	0.924
IFN-γ	18.73 ± 2.43	16.06 ± 1.05	−0.484	0.628	0.894
GM-CSF	3.54 ± 0.38	3.49 ± 0.29	0.093	0.926	0.979
FGF basic (FGF2/bFGF)	11.83 ± 1.72	9.07 ± 1.23	−1.147	0.251	0.774
TSLP	1.14 ± 0.13	1.15 ± 0.05	−1.402	0.161	0.542
ICAM-1 (CD54)	348440.58 ± 70639.64	440232.19 ± 76198.76	−0.464	0.643	0.850
PIGF	2.34 ± 0.18	2.18 ± 0.12	−0.389	0.697	0.860

BH adj, Benjamini-Hochberg adjusted P-value.

There were no significant differences in other cytokines levels including IL-1α, IL-1β, IL-1RA, IL-2, IL-3, IL-4, IL-5, IL-6, IL-7, IL-8, IL-10, IL-12, IL-12 p70, IL-13, IL-15, IL-16, IL-27, IL-31, CCL3, CCL4, CCL11, CCL17, VEGF-C, VEGFR1, TNF-α, Tie-2, IFN-γ, GM-CSF, FGF basic, TSLP, ICAM-1, and PIGF between the two groups (Table 2).

### Association between the group and the abnormal cytokines was evaluated with multiple linear regression analyses

The relationship between the group and the abnormal cytokines was investigated in multiple linear regression models, with adjustments for age, gender, BMI, smoking, and HAMD scores (Table 3). The results revealed that group was a significant independent predictor of serum IL-17C, CCL-26, VEGF, and TNF-β levels, while controlling for other independent variables including age, gender, BMI, smoking, and HAMD-24 scores (all *P* < 0.05). In terms of CXC10, group was also likely to be a significant independent predictor (β = 0.257, *P* = 0.063).

**Table 3:** Association between the group and the abnormal cytokines (multiple linear regression).

**Dependent variables**	**Independent variables**	**β**	***t***	***P***
IL-17C	Group	0.389	2.916	0.005
CCL-26	Group	−0.343	−2.395	0.021
CXC10	Group	0.257	1.905	0.063
VEGF	Group	−0.297	−2.323	0.025
TNF-β	Group	0.305	2.052	0.046

### Correlation between the severity of SI and the cytokines identified as being elevated in MDD patients with SI

The correlation between the severity of suicidal ideation (as measured by the 19-item Beck Scale for Suicide Ideation) and the cytokines identified as being elevated in MDD patients with SI was evaluated by Spearman's correlation test. The results showed that no significant relationship was observed between the severity of SI and serum IL-17C (*r* = −0.159, *P* = 0.409), CXCL10 (*r* = −0.145, *P* = 0.453), and TNF-β (*r* = −0.131, *P* = 0.497) levels in MDD patients with SI.

### Diagnostic values of different cytokines in discriminating MDD patients without SI from MDD patients with SI

The diagnostic performance of different cytokines in discriminating MDD patients with SI from MDD patients without SI were performed by ROC curve analysis (Table 4). Among the 9 different cytokines between the two groups, the AUC values of 2 cytokines including IL-17C and TNF-β were 0.728 and 0.732, respectively.

**Table 4:** ROC analysis of different cytokines in discriminating MDD patients with SI from MDD patients without SI.

**Variables**	**AUC**	***P***	**95% CI**		**Sensitivity**	**Specificity**
			**Lower bound**	**Upper bound**		
IL-17C	0.728	0.005	0.586	0.869	86.2	58.3
CCL26 (Eotaxin-3)	0.698	0.014	0.556	0.841	76.9	53.8
CXCL10 (IP-10/CRG-2)	0.671	0.031	0.526	0.816	69.0	64.0
VEGF	0.679	0.024	0.530	0.827	53.8	85.7
TNF-β (Lymphotoxin)	0.732	0.004	0.593	0.871	65.5	75.0

**Figure 1:**
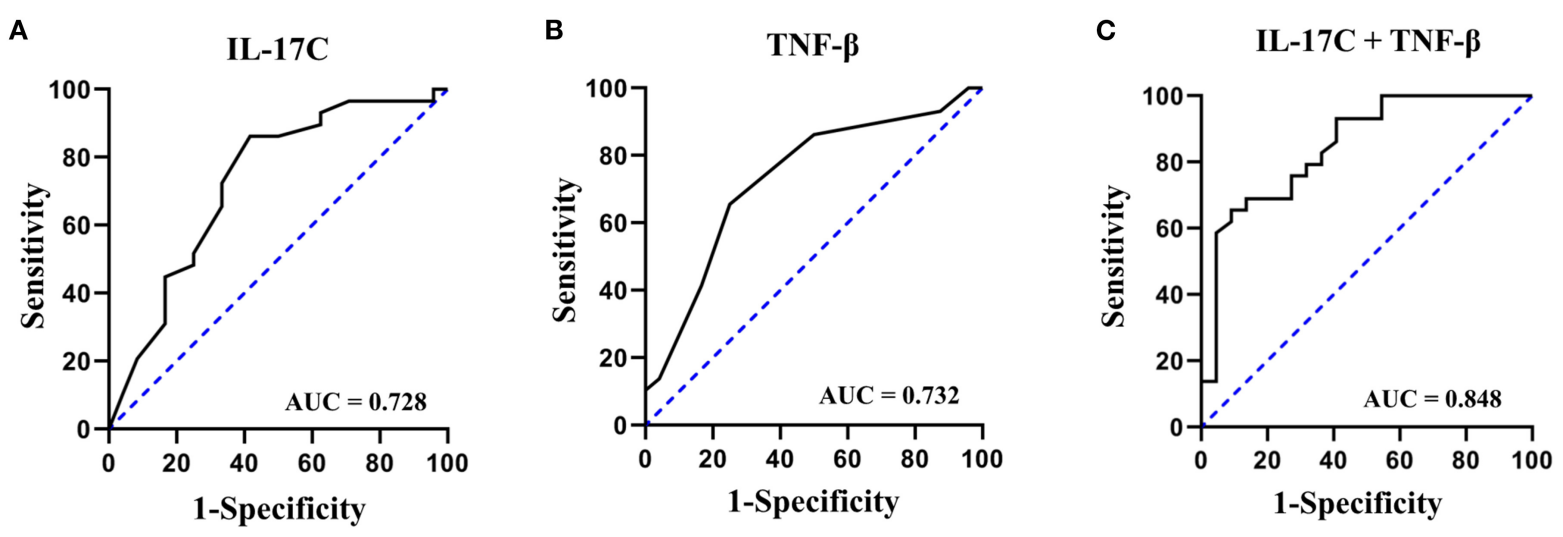
ROC curves of IL-17C, TNF-β, and a combined panel of IL-17C and TNF-β in discriminating MDD patients with SI from MDD patients without SI. **(A)** ROC curve of IL-17C; **(B)** ROC curve of TNF-β; **(C)** ROC curve of a combined panel of IL-17C and TNF-β.

Combining detection of multiple serum proteins as a single panel can improve the sensitivity or specificity of a single biomarker (21). As shown in Figure 1, the ROC curve analysis demonstrated that a combined panel of IL-17C and TNF-β achieved a high accuracy in discriminating MDD patients with SI from MDD patients without SI (AUC = 0.848, sensitivity = 75.9%, specificity = 72.7%).

## Discussion

The present study investigated the serum levels of 37 cytokines in patients with MDD, with the aim to identify serum cytokines-based biomarkers for identification of SI in MDD. Two main findings emerged from the present study. First, MDD patients with SI showed higher levels of IL-17C, CXCL10, and TNF-β, and lower levels of CCL26 and VEGF compared to MDD patients without SI. Second, a combined ROC analysis using IL-17C and TNF-β revealed an AUC of 0.848 with a sensitivity of 75.9% and a specificity of 72.7% in separating MDD patients with SI from MDD patients without SI.

Cytokines are a class of multifunctional proteins involved in cellular communication and activation, that can be used as markers for micro-inflammation (22). Numerous studies have demonstrated the abnormal levels of cytokines in patients with depression. It has been reported that compared to healthy controls, depressed patients exhibited higher levels of IL-6 and TNF-α (23). Further studies have indicated that patients with MDD had increased levels of IL-6, IL-10, IL-12, IL-13, and TNF-α (24). Additionally, abnormal levels of other cytokines including IL-1β, IL-2, IL-4, IL-8, IL-15, CCL3, CCL4, CCL11, and FGF basic have been found in depressed patients in several studies (25–29). These studies focus on evaluating differences in peripheral cytokines between depressed patients and healthy subjects. It is noteworthy that several cytokines including IL-2, IL-6, IL-8, TNF-α and VEGF are reported to be related to the suicidal behavior (17). Early recognition of SI before suicidal behavior in patients with MDD plays an important role in reducing the mortality caused by suicide. Therefore, in the present study, we further compared the differences in serum levels of 37 cytokines between patients without SI and MDD patients with SI. The results confirmed that MDD patients with SI showed higher levels of IL-17C, CXCL10, and TNF-β, and lower levels of CCL26 and VEGF compared to MDD patients without SI. However, after applying the Benjamini-Hochberger correction for multiple measurements, none of these differences persisted. Thus, multicenter large sample studies are required to further confirm these findings. Taken together, these findings link peripheral cytokines to the pathogenesis of depression and SI in depressed patients. Given that this study is a cross-sectional study, the causal relationship between these cytokines and SI needs further studies to explore.

It has been demonstrated that the severity of depressive symptoms may be associated with alterations in cytokine levels (30). Taken together the possible effect of age (31), gender, BMI (32, 33), and smoking (34) on the serum cytokines, the relationship between the group and the abnormal cytokines was investigated in multiple linear regression models, with adjustments for age, gender, BMI, smoking, and HAMD-24 scores. The results revealed that group was a significant independent predictor of serum IL-17C, CCL-26, CXC10, VEGF, and TNF-β levels, further linking these cytokines to the SI of MDD patients. Additionally, there were 3 subjects over 60 years old (2 MDD patients without SI and 1 MDD patients with SI). After we excluded the data of these 3 subjects, the results of this study have not been altered.

We further explored the potential values of these aberrant cytokines as diagnostic biomarkers of SI in MDD. Given that AUC value in ROC analysis should be > 0.7 to be of clinical value for screening (35), IL-17C and TGF-β meet this condition.

IL-17C is a member of the IL-17 family that is selectively induced in epithelia by bacterial challenge and inflammatory stimuli (36). Prior studies showed a crucial role of IL-17C in the pathogenesis of immune-mediated skin diseases (37), autoimmune hepatitis (38), and acute pneumonia (39). To the best of our knowledge, few studies have evaluated the changes in peripheral blood levels of IL-17C in patients with depression. In the present study, we firstly found that the serum concentration of TGF-β was significantly higher in MDD patients with SI compared to MDD patients without SI. Moreover, IL-17C revealed an AUC of 0.728 with a sensitivity of 86.2% and a specificity of 58.3% in separating MDD patients with SI from MDD patients without SI. Since this study is a single-center study and the sample size is relatively small, the aberrant level and the diagnostic value of IL-17C in MDD patients with SI should be confirmed by multicentric studies.

TGF-β is a cytokine with a role in the differentiation of Th17 and T regulatory lymphocytes (40). Previous studies have indicated a crucial role of TGF-β in the pathophysiology of depression. Animal studies have demonstrated an increased TGF-β level in chronic unpredictable mild stress (CUMS) mice, a realistic animal model of depression (41). Moreover, TGF-β was believed to be a key factor responsible for the imbalance between Th17 and Treg cells related to the depression-like behavioral changes in CUMS mice (41). Clinical studies have reported that the TGF-β levels were significantly higher in MDD patients compared to controls (42, 43). After 6 weeks of antidepressant treatment, TGF-β production were significantly lower than before treatment in MDD patients (44). Additionally, the concentration of TGF-β was significantly higher in patients with MDD with childhood maltreatment (CM) history, compared to MDD patients with no CM (45). Combined with the elevated TGF-β levels in MDD patients with SI and the potential diagnostic value of TGF-β in separating MDD patients with SI in the present study, these findings provide more data linking peripheral TGF-β to the disease severity and SI in MDD.

It has been shown that combined detection of multiple serum proteins as a single panel can increase the sensitivity or specificity of a single biomarker (21). Therefore, we further evaluated the potential of a combined panel of IL-17C and TNF-β for the diagnosis of SI in MDD. The results showed that the AUC value of this panel increased to 0.848 with a moderate sensitivity of 75.9% and a moderate specificity of 72.7% in separating MDD patients with SI from MDD patients without SI.

There are some limitations of this study. Firstly, this was a cross-sectional single-center study with a relatively small sample size and the generalizability of the findings was uncertain, which is a significant flaw, and further prospective studies with larger sample sizes are required to confirm our present findings. Secondly, this study is a cross-sectional study, the causal relationship between these cytokines and SI needs further studies to explore. Thirdly, SI was self-reported and may have been subject to reporting bias. Fourthly, the present study only evaluated the SI of MDD patients, not their suicide attempts. As suicide attempt indicates a more severe level of suicidality, further studies are needed to investigate the potential of multiple cytokines as biomarkers for suicide attempt in patients with MDD.

Several public health implications should be highlighted. (1) Given the fact that approximately one million people die of suicide worldwide each year, it is of great clinical significance to identify the patients with SI in a timely and early manner. However, the current diagnosis of SI primarily depends on subjective symptoms/manifestations, with uncertainties as high as 40% as a consequence. Our study for the first time confirms a combined panel of IL-17C and TNF-β for the diagnosis of SI in MDD and may therefore help to identify potentially suicidal patients, especially those patients who are unwilling to disclose suicidal ideation during standard screening. (2) Exercise has been shown to increase growth factor secretion of important cytokines, such as VEGF (46). In the present study, the serum VEGF levels were significantly lower in MDD patients with SI compared to MDD patients without SI. Thus, whether moderate exercise, a public health-related behavior, can improve the SI of MDD patients by increasing the level of blood VEGF needs further studies to investigate. (3) Our results demonstrated that a combined panel of IL-17C and TNF-β achieved a high accuracy in discriminating MDD patients with SI from MDD patients without SI. These two cytokines can be detected by Enzyme-linked immunosorbent assay (ELISA). This detection method is cheap, simple, reliable and fast, which can be set up in all levels of health care facilities including resource limited areas, especially in low to middle income countries.

In conclusion, the present study reveals that MDD patients with SI showed higher levels of IL-17C, CXCL10, and TNF-β, and lower levels of CCL26 and VEGF compared to MDD patients without SI. Our study for the first time confirms a combined panel of IL-17C and TNF-β for the diagnosis of SI in first-episode drug-naïve MDD. These findings provide evidence that alterations in peripheral cytokines levels hold significant promise as biomarkers for identification of SI in MDD.

## Data availability statement

The original contributions presented in the study are included in the article/supplementary material, further inquiries can be directed to the corresponding authors.

## Ethics statement

The studies involving human participants were reviewed and approved by Anhui Mental Health Center. The patients/participants provided their written informed consent to participate in this study.

## Author contributions

YX and QX conceived the study. YX, JG, and QX wrote the protocol. YX, JL, WG, YS, YZ, and FS performed the analyses. YX wrote the first draft. All authors read and commented the manuscript and agreed on the final version.

## Funding

This study was provided by the National Natural Science Foundation of China (81870403), Key Research and Development Program of Anhui Province (202004j07020001), and Hefei Sixth Cycle Key Medical Specialty.

## Conflict of interest

The authors declare that the research was conducted in the absence of any commercial or financial relationships that could be construed as a potential conflict of interest.

## Publisher's note

All claims expressed in this article are solely those of the authors and do not necessarily represent those of their affiliated organizations, or those of the publisher, the editors and the reviewers. Any product that may be evaluated in this article, or claim that may be made by its manufacturer, is not guaranteed or endorsed by the publisher.
